# Balint groups and narrative medicine compared to a control condition in promoting students’ empathy

**DOI:** 10.1186/s12909-020-02316-w

**Published:** 2020-11-09

**Authors:** Cédric Lemogne, Céline Buffel du Vaure, Nicolas Hoertel, Annie Catu-Pinault, Frédéric Limosin, Christian Ghasarossian, Claire Le Jeunne, Philippe Jaury

**Affiliations:** 1 Université de Paris, Faculté de Santé, UFR de Médecine, 15 rue de l’Ecole-de-Médecine, 75006 Paris, France; 2 AP-HP.Centre-Université de Paris, Hôpital Hôtel-Dieu, Service de Psychiatrie de l’adulte, 1 place du parvis Notre-Dame, 75004 Paris, France; 3 Université de Paris, INSERM, Institut de Psychiatrie et Neurosciences de Paris (IPNP), UMR_S1266, 102-108 rue de la Santé, 75014 Paris, France; 4 Université de Paris, Faculté de Santé, UFR de Médecine, Département de Médecine Générale, 24 rue du Faubourg Saint Jacques, 75014 Paris, France; 5 METHODS Team, Epidemiology and Statistics Sorbonne Paris Cité, Research Center UMR 1153, Inserm, 1 place du parvis Notre-Dame, 75004 Paris, France; 6 AP-HP.Centre-Université de Paris, Hôpital Corentin-Celton, Service de Psychiatrie et d’Addictologie de l’adulte et du sujet âgé, 4 parvis Corentin-Celton, 92130 Issy-les-Moulineaux, France; 7 Société Médicale Balint, 10 Route de Thionville, 57140, Woippy, France; 8 AP-HP.Centre-Université de Paris, Hôpital Cochin, Service de Médecine Interne, Paris, France

**Keywords:** Balint groups, Empathy, Medical education, Narrative medicine, Physician-patient relations, Students, medical

## Abstract

**Background:**

The perceived importance of clinical empathy may decline among students during medical training. Several interventions have been shown to be effective in promoting or preserving medical students’ empathic abilities, such as empathy skills training or Balint groups. Although narrative medicine training shares some features with these interventions, no randomized study to date examined the efficacy of narrative medicine training. This study aimed to assess the effects of Balint groups and narrative medicine training on clinical empathy measured by the self-rated Jefferson’s School Empathy Scale - Medical Student (JSPE-MS©) among fourth-year medical students.

**Methods:**

Students who gave their consent to participate were randomly allocated in equal proportion to Balint groups, narrative medicine training or to the control group. Participants in the intervention groups received either seven sessions of 1.5-h Balint groups or a 2-h lecture and five sessions of 1.5-h narrative medicine training from October 2015 to December 2015. The main outcome was the change in JSPE-MS© score from baseline to one week after the last session.

**Results:**

Data from 362 out of 392 participants were analyzed: 117 in the control group, 125 in the Balint group and 120 in the narrative medicine group. The change in JSPE-MS© score from baseline to follow-up was significantly higher in the Balint group than in the control group [mean (SD): 0.27 (8.00) vs. -2,36 (11.41), *t* = 2.086, *P* = 0.038]. The change in JSPE-MS© score in the narrative medicine group [mean (SD): − 0.57 (8.76)] did not significantly differ from the changes in the control group (*t* = 1.355, *P* = 0.18) or the Balint group (*t* = 0.784, *P* = 0.43). Adjusting for participants’ characteristics at baseline, Balint groups remained associated with better outcomes compared to the control group (β = 2.673, *P* = 0.030).

**Conclusions:**

Balint groups may promote clinical empathy to some extent among medical students, at least in the short run.

## Background

Empathy, which can be defined as the ability to share and/or understand others’ emotional state without confusion between self and others, is considered as a core feature of the doctor-patient relationship [1, 2]. In addition to these affective and cognitive component, clinical empathy, i.e. empathy within the context of a doctor-patient relationship, also encompasses motivational and behavioral components, corresponding to the way the doctor acts according to the patient’s emotional state and expresses his or her understanding of that state. In accordance, most of medical schools emphasize the need to promote clinical empathy during the medical students’ curriculum [3]. The potential benefit of clinical empathy may go far beyond patients’ satisfaction and extend to better clinical assessment, therapeutic alliance and compliance, and thus better clinical outcome [4, 5]. Unfortunately, several cross-sectional and longitudinal studies have documented a weak, yet significant decline of the empathic abilities during medical training [6–8].

In order to promote medical students’ empathic abilities, several interventions have been proposed, such as empathy skills training [9, 10], narrative medicine [11–13] or Balint groups [14, 15]. These interventions build on the hypothesis that at least some of the four above-mentioned components of clinical empathy (i.e. affective, cognitive, motivational and behavioral) are teachable. For instance, there is evidence that the behavioral component of clinical empathy (i.e. “showing empathy”) may be enhanced in certified physicians by communication skills training [16], whereas the cognitive component (i.e. “putting oneself in the patient’s shoes”) may be promoted in medical students when they are invited to write the patient’s story from a first-person perspective during narrative medicine training [12]. Other interventions, such as Balint groups, are thought to foster several components of clinical empathy through a variety of pathways, including not only the growth of participants’ reflexivity, but also the implicit promotion of humanistic values [14, 15]. A recent meta-analysis of randomized controlled trials of clinical empathy interventions in medical students included 16 studies aiming at promoting empathy through didactic, experiential or behavioral (i.e. skills training) approaches. Overall, this meta-analysis found a moderately positive effect on clinical empathy after an intervention compared to control conditions, but heterogeneity was high and type of intervention was a significant effect modifier [17]. While purely didactic interventions were not effective, interventions combining different approaches were the most effective, following by skills training – especially when rehearsal was present – and experiential training, including Balint groups. The combination of various intervention types with various types of empathy measure was also a source of heterogeneity. For instance, in a trial by Wûndrich et al. [9], students who were offered an empathy skills training with simulated patients (vs. a control condition) showed significantly higher levels of empathy after the intervention when rated with an Objective Structured Clinical Examination (OSCE) but not when self-rated with the Jefferson’s School Empathy Scale - Medical Student (JSPE-MS©) [7]. In contrast, another randomized trial conducted by our group [15] found seven sessions of 1.5-h Balint groups (vs. a control condition) to be associated with an improvement of the JSPE-MS© score one week after the last session, but not in empathy measured by standardized patients during OSCE. Although the extent to which the components that are theoretically targeted by a specific intervention are actually and specifically modified remains unclear, such differences suggest that different interventions may target different components of empathy.

Building on data collected during our two-site randomized study that compared Balint groups with a control condition [15], we took advantage of another intervention group implemented in one site only in which students were offered a narrative medicine training and an assessment of clinical empathy with the JSPE-MS©. Our aim was to compare Balint groups and narrative medicine training with a control condition. A Balint group is a group of clinicians, often physicians, who meet regularly to present clinical cases in order to improve and to better understand the clinician-patient relationship. Balint groups are specifically designed to help health-professionals and medical students in reducing interpersonal difficulties while taking into account emotional issues [18–20]. Narrative medicine encourages physicians to consider patients’ narratives in clinical practice and to engage in self-reflection [11–13]. Compared to the control group, we hypothesized that both Balint groups and narrative medicine training would result in increased JSPE-MS© scores from baseline to follow-up.

## Methods

### Setting and participants

The original study was conducted from October 2015 to December 2015 at Paris Diderot University and Paris Descartes University (Paris, France). Eligible students were fourth-year medical students who gave their consent to participate through a secured website. Since narrative medicine training was not randomly allocated at Paris Diderot University, only data from Paris Descartes University were considered in the present analysis.

In France, medical education involves six years of medical school before internship. The present study was proposed to all the students at the beginning of their fourth-year curriculum (N = 392). There were no exclusion criteria. The study obtained ethical approval from the Institutional Review Board of Paris Descartes University, Paris, France (number 00001072).

### Group allocation

The fourth-year curriculum includes three consecutive 3-month thematic learning programs: program A (cardiology, respiratory medicine, thoracic surgery, etc.), program B (gastroenterology, endocrinology, gastrointestinal surgery, etc.), and program C (internal medicine, rheumatology, orthopedic surgery, etc.). Each program involves practical learning in the morning through clerkship at the hospital, and theoretical learning in the afternoon through small group tutorials or lectures at the faculty. Within each program, both practical and theoretical learnings have thematically related contents so that the students can take advantage of the clerkship to apply their theoretical knowledge to real clinical situations. At the end of each consecutive 3-month program, the students’ validation is based on a theoretical exam at the faculty and an assessment of the hospital clerkship by the supervisors. At the beginning of each year, each student is randomly allocated to one of three groups (1:1:1) by the Paris Descartes University staff using a locally developed software: group 1 follows program A first, then B and C; group 2 follows program B first, then C and A; and group 3 follows program C first, then A and B. During the study period, program B included narrative medicine training and program C included Balint groups, while program A did not include any teaching related to clinical empathy. In the present study, the students randomly allocated to group 1, 2 and 3 were thus considered as participants allocated to the control condition, narrative medicine training, and Balint groups, respectively. This randomization is routinely performed each year and the investigators of the present study had no role in the allocation of the students. They were only involved in inviting students to participate to the study (i.e. data collection as detailed below) through a secured website.

### Intervention

Students allocated to the Balint groups were randomly split into groups of 12 or 13 students. Each group received a training over two months that included seven weekly 1.5-h Balint group sessions. Participants in Balint groups were asked to react to a particularly touching, upsetting or interesting lived clinical situation that involves interpersonal problems, under the supervision of a trained facilitator [14]. This training was not specifically tailored to improve empathy and did not differ from usual Balint group sessions. Before the beginning of the study, all the facilitators were accredited as Balint groups’ leaders either by the French Balint Medical Society (Société Médicale Balint France) or the Balint Training Association (Association de Formation Balint). To homogenize the intervention, regular meetings among facilitators were organized before and during the study.

Students allocated to the narrative medicine training first received a 2-h lecture introducing the theoretical background of narrative medicine and clinical empathy. Then, they were randomly split into groups of 9 or 10 students and received a training over two months that included five weekly 1.5-h sessions of reflective reading and writing. In addition, five hours were devoted to homework. To homogenize the intervention, regular meetings among teachers were organized before and during the study.

Students included in the control group received no specific training.

### Procedure

Baseline characteristics were self-reported and included gender, education level of the most educated parent (primary / secondary / undergraduate or graduate / postgraduate), living status (alone, with parents, or other), anticipated specialty choice (surgery, medicine, other) [21]. In addition, all participants had to complete the validated French version of the JSPE-MS© [22] at baseline through a secured website. This scale encompasses 20 Likert-type items, rated from 1 (strongly disagree) to 7 (strongly agree) (e.g. “Patients feel better when their physicians understand their feelings”), leading to a summed score ranging from 20 to 140 with higher score indicating higher levels of empathy.

One week after the last session, all participants had to complete the JSPE-MS© again.

### Blinding

Whereas students and facilitators were aware of the allocated group, data analysts were kept blinded to the allocation.

### Statistical analysis

Based on the results of our larger two-site study, which found a between-group difference of 4.2 points with a standard deviation (SD) of 11.7 points regarding the JSPE-MS© score [15], we calculated that 124 participants per group would be needed to ensure a significance level of 5% and a statistical power of 80% [23] in order to detect a difference between one of the intervention groups (i.e. Balint groups and narrative medicine training) and the control group, thus allowing the present analyses to be conducted.

Because of a technical problem, data were systemically missing for one item of the JSPE-MS© (i.e. “Physicians should try to stand in their patients’ shoes when providing care to them”). However, the internal consistency in our sample remained good (Cronbach’s alpha: 0.77 and 0.80 for the baseline and follow-up measures, respectively) and thus allowed computing a one-dimension global score based on the mean value of the 19 available items multiplied by 20.

Descriptive results were reported with means and SDs for quantitative variables or absolute frequencies and percentages by modality for qualitative variables. Between-group differences regarding the covariates and the outcome (i.e. the change in JSPE-MS© score from baseline to one week after the last session) were tested with ANOVAs and Student’s t-tests for continuous variables and Chi-square tests for categorical variables. Statistical significance was evaluated using a two-sided design with alpha set a priori at 0.05. A moderating role of gender was searched for by including the group, the gender and the group by gender interaction term simultaneously in a general linear model.

To test the robustness of our results, a multivariate analysis used a general linear model with the change in JSPE-MS© score from baseline to follow-up as the dependent variable and all participants’ characteristics at baseline as independent variables.

All statistical analyses were computed using SPSS 16.0.1 software (SPSS Inc., Chicago, IL, USA).

## Results

**Fig. 1:**
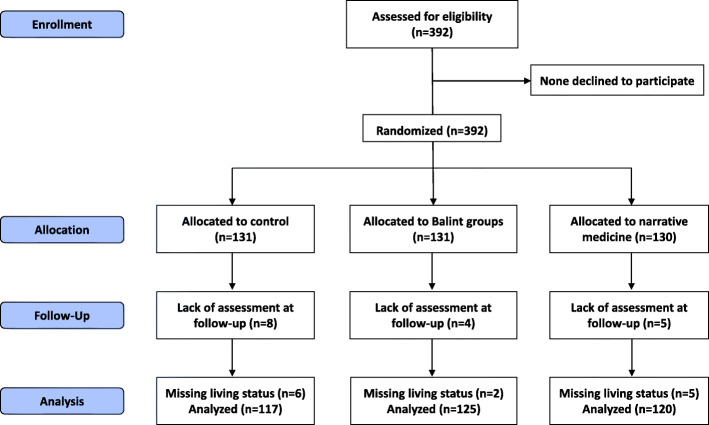
Flow diagram of the present study

Among the 392 students who were randomized, 17 did not complete the JSPE-MS© at follow-up and 13 had missing data regarding living status. This resulted in a final sample of 362 participants, including 117 in the control group, 125 in the Balint group and 120 in the narrative medicine group (Fig. 1 – Flow diagram). There was no association between group allocation and JSPE-MS© completion at follow-up (*χ*^2^ = 1.585, *P* = 0.45). The three groups did not differ regarding socio-demographic factors, specialty choice or JSPE-MS© score at baseline (Table 1). Missing JSPE-MS© score at baseline (N = 4) were then replaced with the mean value of the study population. The two intervention groups did not differ regarding the number of absences for Balint groups or narrative medicine sessions of reflective reading and writing (Table 1).

**Table 1:** Participants’ characteristics at baseline according to group allocation, Paris Descartes University, October to December 2015

	Control (***N*** = 117)		Balint groups (***N*** = 125)		Narrative medicine (***N*** = 120)			
**Discrete variables**	**N**	**%**	**N**	**%**	**N**	**%**	**χ**^**2**^	***P***
***Gender***							2.148	0.34
Female	75	64.1	72	57.6	66	55.0		
Male	42	35.9	53	42.4	54	45.0		
***Parental education level***^***a***^							1.854	0.40
Undergraduate	7	6.0	10	8.0	13	10.8		
Graduate / postgraduate	110	94.0	115	92.0	107	89.2		
***Living status***							9.493	0.050
Alone	27	23.1	40	32.0	44	36.7		
With parents	57	48.7	56	44.8	59	49.2		
Neither alone nor with parents	33	28.2	29	23.2	17	14.2		
***Specialty choice***							5.596	0.23
Surgery	24	20.5	16	12.8	27	22.5		
Medicine	82	70.1	94	75.2	79	65.8		
Other	11	9.4	15	12.0	14	11.7		
***Number of absences***							3.323	0.19
No absence	–	–	69	55.2	74	61.7		
One absence	–	–	42	33.6	40	33.3		
More than one absence	–	–	14	11.2	6	5.0		
**Continuous variables**	**Mean**	**SD**	**Mean**	**SD**	**Mean**	**SD**	***F***	***P***
***JSPE-MS© score***								
Full sample	110.1	11.9	111.0	9.1	110.7	9.3	0.277	0.76
Female	111.8	11.8	112.3	8.6	113.0	8.0	0.270	0.76
Male	107.0	11.5	109.3	9.5	108.0	10.2	0.594	0.55

^a^education level of the most educated parent

*JSPE-MS©* Jefferson Scale of Physician Empathy for Medical Students

The change in JSPE-MS© score from baseline to follow-up was significantly higher in the Balint group than in the control group [mean (SD): 0.27 (8.00) vs. -2,36 (11.41), *t* = 2.086, *P* = 0.038]. The change in JSPE-MS© score in the narrative medicine group [mean (SD): − 0.57 (8.76)] did not significantly differ from the changes observed in the control group (*t* = 1.354, *P* = 0.18) or the Balint group (*t* = 0.784, *P* = 0.43). Among the Balint group, there was no significant difference regarding changes in JSPE-MS© scores from baseline to follow-up between participants who did not miss any session [0.88 (7.67), *N* = 69], those who missed one session [0.15 (8.42), *N* = 42] or more than one [− 2.41 (8.28), *N* = 14] (*F* = 0.993, *P* = 0.37). The group by gender interaction was not significant (*F* = 1.099, *P* = 0.33), which prevented further stratified analyses.

To test the robustness of our results, a general linear model took into account all participants’ characteristics and found Balint groups associated with more favorable changes in JSPE-MS© score compared to the control group (Table 2).

**Table 2:** General linear model predicting the change in JSPE-MS© score from baseline to one week after the last session

Term	β	95% confidence interval	SE	***t***	***P***
Constant	−5.142	−8.187, −2.097	1.548	3.321	0.001
Balint groups	2.673	0.258, 5.088	1.228	2.176	0.030
Narrative medicine	2.181	−0.280, 4.641	1.251	1.743	0.082
Female gender	1.455	−0.553, 3.463	1.021	1.425	0.155
Undergraduate parental education	1.672	−1.901, 5.246	1.817	0.920	0.358
Living alone	−0.191	−2.477, 2.095	1.162	0.165	0.869
Living neither alone nor with parents	2.098	−0.452, 4.649	1.297	1.618	0.107
Surgery choice	1.835	−1.897, 5.566	1.897	0.967	0.334
Other specialty choice	1.470	−1.125, 4.066	1.320	1.114	0.266

Reference categories for discrete variables were control group, male gender, graduate / postgraduate parental education, living with parents and medicine choice

*β* estimated coefficient, *JSPE-MS©* Jefferson Scale of Physician Empathy for Medical Students, *SE* Standard Error

## Discussion

The present randomized study aimed to assess the efficacy of Balint groups or narrative medicine training to promote clinical empathy among medical students. Compared to a control group, participants allocated to the Balint groups showed more favorable changes in self-reported clinical empathy. Participants allocated to narrative medicine training had in-between outcomes, which did not differ from those observed in the Balint group or in the control group. When accounting for baseline participants’ characteristics in a multivariate analysis, Balint groups remained associated with better outcomes compared to the control group, while there was a similar trend, yet not significant for the narrative medicine group.

To our knowledge, this is the first randomized study to examine the efficacy of narrative medicine training in promoting empathy during medical school. This study has several strengths, including the random allocation to intervention or control groups and the use of a validated self-report measure of clinical empathy. Since empathy is critically modulated by contextual factors [24], it is noteworthy that this measure focused on empathy in the context of the doctor-patient relationship. Despite smaller sample sizes, the present results suggesting a positive effect of Balint groups on the clinical empathy of fourth-year medical students of Paris Descartes University are consistent with the lack of significant site by intervention interaction observed in our previous report, thus supporting the homogeneity of the effect across study sites [15]. Since 111 (89%) students allocated to Balint groups missed no more than one of the seven sessions, we could not show a significant association between attendance and changes in self-reported empathy, but this suggests that this intervention was well-accepted, as was narrative medicine training.

Some limitations should be acknowledged. First, although the sample size was large enough to compare the intervention groups (i.e. Balint groups or narrative medicine training) vs. the control group, our study was underpowered to draw conclusions as regards the lack of difference between the two intervention groups. Second, empathy was not measured by standardized patients during OSCE in the narrative group as it was in the Balint and control groups, thus restricting the between-group comparisons to self-reported clinical empathy. In addition, self-reported measures may be more influenced by social desirability biases than objective measures. Third, the effect size of the Balint groups intervention was rather small and could be of limited duration after the end of the training. More specifically, the clinical significance of the mean difference in the JSPE-MS© score at follow-up between participants allocated to the Balint groups and those allocated to the control condition (i.e. 2.4 points) is unclear. For instance, this difference is roughly equivalent to the one-third of the difference that was observed between students at the end of their second year vs. those of the end of their third year in the seminal study by Hojat et al. [7]. However, Balint groups are usually implemented over a longer period, which may thus result in greater effect size in the long run. Finally, the three groups differed regarding the exposure to hospital clerkship or other learning programs (e.g. cardiology, rheumatology) so our study may not be formally considered as a ‘controlled’ study despite randomization. Although none of these programs specifically relates to empathy or doctor-patient relationship, we cannot rule out the hypothesis that they might partially account for the present results.

The so-called decline of empathy during medical school may be more likely to be a decline of the value given to empathy, rather than a decline of empathic abilities per se [25–27]. Significant models (e.g., senior physicians) [28], teaching methods [29] or selection procedures [30] may contribute to this decline. Coping strategies based on emotional distancing [31] may also account for this decline. Interestingly, Balint groups may both promote empathy as a value shared by the medical community and modulate self-distancing by providing medical students with other tools to regulate stressful emotions elicited by clinical practice [18, 32]. For instance, perspective-taking may protect physicians from such detrimental effects while allowing them to show sustained empathic concern [33]. Likewise, narrative medicine training includes some exercises such as encouraging students to tell the story of a patient from his or her own, first-person perspective or getting acquainted with patient-written narratives that may foster clinical empathy by both promoting humanistic values and perspective-taking. Eventually, narrative medicine training is also thought to enable physicians recognizing “their own personal journeys through medicine, acknowledging kinship with and duties toward other health care professionals” [11]. Since the promotion of empathy and self-reflection is at core of narrative medicine and based on evidence from a pre-post observational study [13], we hypothesized that this intervention would also result in higher JSPE-MS© score changes from baseline to follow-up than in control group. Although the difference was not statistically significant, it was nonetheless in the expected direction and close to significance in the multivariate analysis.

## Conclusions

The present study suggests that Balint groups may contribute to promote, or at least preserve, clinical empathy among medical students, as previously found in a larger setting [15], but failed to provide some support for a similar effect of narrative medicine. Further studies are needed to refine our understanding of the components that may be shared by these two kinds of teaching in order to develop more efficient interventions. For instance, these studies may include experimental manipulation of some components of the interventions as well as qualitative studies exploring the narratives of students exposed to these interventions. In addition, long-term studies are needed to explore both the effects of a short intervention in the long run and the effects of longer interventions.

## Data Availability

The datasets used and/or analyzed during the current study are available from the corresponding author on reasonable request.

## Abbreviations

JSPE-MS©: Jefferson Scale of Physician Empathy for Medical Students
OSCE: Objective Structured Clinical Examination
SD: Standard Deviation
SE: Standard Error

## References

[1] Kelm Z, Womer J, Walter JK, Feudtner C (2014). Interventions to cultivate physician empathy: a systematic review. BMC Med Educ.

[2] Hemmerdinger JM, Stoddart SDR, Lilford RJ (2007). A systematic review of tests of empathy in medicine. BMC Med Educ.

[3] Learning objectives for medical student education--guidelines for medical schools: report I of the Medical School Objectives Project. Acad Med J Assoc Am Med Coll. 1999;74(1):13–8. https://pubmed-ncbi-nlm-nih-gov.proxy.insermbiblio.inist.fr/9934288/. 10.1097/00001888-199901000-00010 9934288

[4] Hojat M, Louis DZ, Markham FW, Wender R, Rabinowitz C, Gonnella JS (2011). Physicians’ empathy and clinical outcomes for diabetic patients. Acad Med J Assoc Am Med Coll.

[5] Del Canale S, Louis DZ, Maio V, Wang X, Rossi G, Hojat M (2012). The relationship between physician empathy and disease complications: an empirical study of primary care physicians and their diabetic patients in Parma, Italy. Acad Med J Assoc Am Med Coll.

[6] Neumann M, Edelhäuser F, Tauschel D, Fischer MR, Wirtz M, Woopen C (2011). Empathy decline and its reasons: a systematic review of studies with medical students and residents. Acad Med J Assoc Am Med Coll.

[7] Hojat M, Vergare MJ, Maxwell K, Brainard G, Herrine SK, Isenberg GA (2009). The devil is in the third year: a longitudinal study of erosion of empathy in medical school. Acad Med J Assoc Am Med Coll.

[8] Colliver JA, Conlee MJ, Verhulst SJ, Dorsey JK (2010). Reports of the decline of empathy during medical education are greatly exaggerated: a reexamination of the research. Acad Med J Assoc Am Med Coll.

[9] Wündrich M, Schwartz C, Feige B, Lemper D, Nissen C, Voderholzer U (2017). Empathy training in medical students - a randomized controlled trial. Med Teach.

[10] Riess H, Kelley JM, Bailey RW, Dunn EJ, Phillips M (2012). Empathy training for resident physicians: a randomized controlled trial of a neuroscience-informed curriculum. J Gen Intern Med.

[11] Charon R (2001). Narrative Medicine: A Model for Empathy, Reflection, Profession, and Trust. JAMA.

[12] Goupy F, Abgrall-Barbry G, Aslangul E, Chahwakilian A, Delaitre D, Girard T (2013). Can narrative medicine be an answer to patient physician relationship teaching according to students’ demand in medical education curricula?. Presse Med.

[13] Chen P-J, Huang C-D, Yeh S-J (2017). Impact of a narrative medicine programme on healthcare providers’ empathy scores over time. BMC Med Educ.

[14] Airagnes G, Consoli SM, De Morlhon O, Galliot A-M, Lemogne C, Jaury P (2014). Appropriate training based on Balint groups can improve the empathic abilities of medical students: a preliminary study. J Psychosom Res.

[15] Buffel du Vaure C, Lemogne C, Bunge L, Catu-Pinault A, Hoertel N, Ghasarossian C (2017). Promoting empathy among medical students: A two-site randomized controlled study. J Psychosom Res.

[16] Moore PM, Rivera S, Bravo-Soto GA, Olivares C, Lawrie TA (2018). Communication skills training for healthcare professionals working with people who have cancer. Cochrane Database Syst Rev.

[17] Fragkos KC, Crampton PES. The Effectiveness of Teaching Clinical Empathy to Medical Students: A Systematic Review and Meta-Analysis of Randomized Controlled Trials. Acad Med. 2020;95(6):947–57. 10.1097/ACM.0000000000003058 31688037

[18] Freyberger H, Besser L (1982). Teaching psychosomatic medicine with special reference to the Balint group and the case supervision group. Psychother Psychosom.

[19] Torppa MA, Makkonen E, Mårtenson C, Pitkälä KH (2008). A qualitative analysis of student Balint groups in medical education: contexts and triggers of case presentations and discussion themes. Patient Educ Couns.

[20] Van Roy K, Vanheule S, Inslegers R (2015). Research on Balint groups: a literature review. Patient Educ Couns.

[21] Rotge J-Y, Lemogne C, Jouvent R, Fossati P (2015). Relationship between personality dimensions and medical specialty in 1661 residents. J Psychosom Res.

[22] Bitoun A (2017). Validation du score JSPE-Medical Student© en langue française dans l’évaluation de l’empathie des étudiants en médecine (MD dissertation).

[23] Norman G, Streiner D (1999). PDQ statistics.

[24] Decety J, Jackson PL (2004). The functional architecture of human empathy. Behav Cogn Neurosci Rev.

[25] Pedersen R (2009). Empirical research on empathy in medicine-a critical review. Patient Educ Couns.

[26] Lemogne C (2015). Empathy in medicine, necessary but not free from risks. Rev Prat.

[27] Smith KE, Norman GJ, Decety J (2017). The complexity of empathy during medical school training: evidence for positive changes. Med Educ.

[28] Newton BW, Barber L, Clardy J, Cleveland E, O’Sullivan P (2008). Is there hardening of the heart during medical school?. Acad Med J Assoc Am Med Coll.

[29] Garden R (2009). Expanding clinical empathy: an activist perspective. J Gen Intern Med.

[30] Chen DCR, Kirshenbaum DS, Yan J, Kirshenbaum E, Aseltine RH (2012). Characterizing changes in student empathy throughout medical school. Med Teach.

[31] Halpern J (2003). What is clinical empathy?. J Gen Intern Med.

[32] Turner AL, Malm RL (2004). A preliminary investigation of balint and non-balint behavioral medicine training. Fam Med.

[33] Gleichgerrcht E, Decety J (2013). Empathy in clinical practice: how individual dispositions, gender, and experience moderate empathic concern, burnout, and emotional distress in physicians. PLoS One.

